# The Mediating Role of Psychological Capital on the Association between Occupational Stress and Job Satisfaction among Township Cadres in a Specific Province of China: A Cross-Sectional Study

**DOI:** 10.3390/ijerph14090972

**Published:** 2017-08-28

**Authors:** Chang-Yue Shang Guan, Yu Li, Hong-Lin Ma

**Affiliations:** Department of Epidemiology and Health Statistics, School of Public Health, Jinzhou Medical University, No. 40, Section 3, Songpo Road, Linghe District, Jinzhou 121001, Liaoning, China; shangguanyp@163.com (C.-Y.S.G.); 15841666337@163.com (Y.L.)

**Keywords:** occupational stress, psychological capital, job satisfaction, township cadres

## Abstract

*Background*: Township cadres, considered as basic executors of state policy, play an important role in Chinese society. Their job satisfaction is a vital issue for township management, but there are few studies on this topic in China. The goal of this study is to analyze the relationship between occupational stress and job satisfaction, and to further examine whether psychological capital (PsyCap) can serve as a mediator between stress and job satisfaction in Chinese township cadres. *Methods*: A cross-sectional survey was carried out during the period of from October 2015 to January 2016 in Liaoning Province of China. The questionnaires, which consisted of an effort-reward imbalance scale, Minnesota Satisfaction Questionnaire (MSQ) for job satisfaction, and the psychological capital questionnaire (PCQ-24), as well as questions about demographic characteristics, were distributed to 1800 township cadres and complete responses were received from 1525 participants. Structural equation modeling was used to examine the role that psychological capital played in mediating between occupational stress and job satisfaction. *Results*: In the present study, effort-reward ratio (ERR= 11 × effort/6 × reward) was negatively associated with job satisfaction (r = −0.372, *p* < 0.001), whereas psychological capital was positively associated with job satisfaction in township cadres (r = 0.587, *p* < 0.001) from a specific province in China. Psychological capital is a mediator between the association of job stress and job satisfaction. *Conclusions*: Psychological capital partially mediated the relationship between job stress and job satisfaction among Chinese township cadres. Interventions to improve Chinese township cadres’ job satisfaction should be developed in the future, especially the enhancement of PsyCap. Interventions need to be verified in further cohort studies. At present, we are only proposing a theoretical model. Intervention effects need to be validated in further cohort studies.

## 1. Introduction

In China, the government comprises the following components: central, provincial (autonomous regions and municipalities directly under the central government), prefecture-level cities (districts), county, and townships. Township governments constitute the basic state administrative organization and area critical bridge between the state and the people. It is generally believed that township cadres, regarded as basic executors of state policy, play an important role in society. Their job performance directly influences the effectiveness of party and government policies, the perceived public reputation of the government, the country’s economic development, and the advancement of socialist modernization [1]. The work of civil servants can improve the competitiveness and overall strength of the country. Previous research has shown that the job satisfaction of civil servants directly affects their government performance levels [2].

Occupational stress usually refers to physical and mental health pressures, and body function disorders, due to the imbalance between staff ability and their objective demands [3]. Some scholars utilize an effort-reward imbalance model (ERI) to assess occupational stress. This model focuses on the reciprocity of extrinsic and intrinsic effort with reward [4]. Studies have reported that an effort-reward imbalance, along with overcommitment, significantly predicts negative outcomes, such as mental disorders, immune system dysfunction, and low job satisfaction, in a variety of professions [5,6,7]. Previous studies have shown that leading township cadres suffered from increased mental health problems, high stress and anxiety due to increasing workload, high social expectations, low political treatment, slow career development, low economic outcomes, combined with a monotonous life, child rearing and health pressures [8,9,10]. Moreover, one study revealed that 87.5% of township cadres experienced some extent of anxiety and psychological distress [11]. Empirical studies have found that occupational stress negatively predicts levels of job satisfaction [12,13]. However, as far as we know, no study has explored the relationship between occupational stress and job satisfaction among township cadres in China.

Job satisfaction can be defined as a subjective feeling of how well one’s needs are being met by their job, or as “the extent to which people like their jobs” [14]. Job satisfaction is influenced by culture, individual factors, and organizational environment. Meanwhile, job satisfaction reflects one’s satisfaction with the organization to a certain extent; that is, if an individual is satisfied with the organization, he or she might exhibit increased performance [14]. In America, a recent study found that improved job satisfaction can increase staff awareness of unit performance [15]. Similarly, in China, investigators have reported that job satisfaction may represent an appropriate indicator of employee productivity and commitment to an organization [16,17]. In a study from England that included multiple occupations, a lower level of job satisfaction was found to be associated with increased staff turnover. Turnover results in inferior job performance and negatively impacts quality of care for residents [18]. Therefore, examining job satisfaction can not only increase the ability of reformation, but it can also improve job-related behaviors, job performance, turnover reduction, and absenteeism [19,20]. In China, empirical studies have shown that township cadres are put under comprehensive pressure that includes substantial work-related responsibilities and poor working environment, which can contribute to mental health problems and job stress [10,21]. Studies show that working environment, reward, and anxiety are all associated with job satisfaction among township cadres [22,23]. Moreover, studies in various populations suggest that job satisfaction is associated with burnout, rewards, work-related stress and social anxiety [24,25,26].

A previous study has attempted to identify positive resources, such as psychological capital (PsyCap), for combating occupational stress [7]. PsyCap is recognized as a condition of positive psychological capacities that include self-efficacy, hope, optimism, and resilience, which can be measured, developed, and changed with various outcomes [27]. In other words, individuals with high PsyCap values can effectively deal with problems, anticipate good results, recover quickly from frustration, and confront negative situations with a better attitude. The positive effects of PsyCap have been explored in various occupations, such as nursing, teaching and law enforcement [28,29,30]. Studies have also demonstrated a positive correlation between job performance and satisfaction, a feeling of well-being, and PsyCap [27,31,32]. In addition, the function of PsyCap as a mediator has attracted the attention of many researchers in various fields, including medicine and education. For instance, a study reported that PsyCap functioned as a mediator between work-family and burnout among female Chinese nurses [33]. Wang and colleagues also suggested that PsyCap mediates the relationship between job satisfaction and performance [31]. The findings of Lu et al. demonstrate that PsyCap mediates the relationship between the imbalance of effort-reward and job satisfaction among Chinese police [7]. In the current social environment, it is very difficult to improve job satisfaction via changing the situation of occupational stress completely. Therefore, we hypothesize that PsyCap might play a role in mediating the relationship between occupational stress and job satisfaction among Chinese township leaders. Up to this point, this hypothesis has not been investigated.

Our study aimed to assess job satisfaction among Chinese township cadres and to explore the association between occupational stress, job satisfaction and PsyCap. Meanwhile, the role of PsyCap in mediating the associations between job satisfaction and occupational stress was also examined.

## 2. Methods

### 2.1. Study Design and Sample

A cross-sectional survey was conducted from October 2015 to January 2016 on a population of township cadres in Liaoning Province of China. Given the geographic division and economic development in Liaoning Province, it was divided into four regions (western, southern, northern, eastern). One city was randomly selected from each geographic region. Ten townships were randomly selected within each sampled city. If the sampled city was a megalopolis, three additional townships were randomly selected. As a result, a total of 46 townships and 1800 township cadres were recruited for this study. After written informed consent was obtained to conduct this study, a set of self-administered questionnaires was distributed to the township cadres, which were completed anonymously. Eventually, a pool of 1525 participants (effective response rate: 84.72%, 316 in eastern, 451 in southern, 345 in western, 413 in northern) became the potential study sample. The study was approved by the Ethics Committee of Jin Zhou Medical University, and the study procedures were in accordance with ethical standards.

### 2.2. Demographic Characteristics

Information on gender, age, educational level, marital status, monthly income and years of service in the township public institution were collected in this study.

### 2.3. Measurement of Job Satisfaction

Job satisfaction was measured using a shorter Chinese version of the Minnesota Satisfaction Questionnaire (MSQ) [34]. This version is a 20-item short form with each item scored on a 5-point Likert scale ranging from 1 (very dissatisfied) to 5 (very satisfied) and it included three dimensions: intrinsic job satisfaction, extrinsic job satisfaction, and general job satisfaction. Intrinsic job satisfaction includes 12 items that covers activity, ability utilization, achievement, and so forth. Extrinsic job satisfaction includes six items that refer to supervisor-human relations, compensation, company policies, compensation, and so forth. Higher scores reflect a higher level of job satisfaction. Overall satisfaction is demonstrated by the total score of all 20 items, which ranges from 20 to 100. A score of 60 indicates a neutral attitude, a score ranging from 61 to 79 suggests moderate satisfaction, and a score of 80 indicates high satisfaction [35]. The Cronbach’s alpha coefficients for the overall job satisfaction, intrinsic job satisfaction and extrinsic job satisfaction subscales were 0.928, 0.899, and 0.836, respectively.

### 2.4. Measurement of Occupational Stress

The effort-reward imbalance (ERI) model was used to assess the level of occupational stress among township cadres. The Chinese version of the ERI has been widely used with good reliability in China [36]. The 23-item ERI questionnaire consists of three dimensions, including extrinsic effort (six items), reward (11 items), and overcommitment (six items). The effort and reward were measured using two steps: first, participants must agree or disagree to the attitude of their work conditions described in the item; and second, if they choose the answer “disagree” regarding their work conditions, they then have to describe the degree to which they feel distressed. The response to questions that address effort and reward were scored on a five-point scale (ranging from 1 = not stressful to 5 = very stressful). Overcommitment was scaled from 1 to 4, in which 1 indicates strong disagreementand 4 indicates strong agreement, with higher scores indicating increasing overcommitment to work. The extent of job stress (the ERI scale) can be expressed by the effort-reward ratio (ERR) and overcommitment, respectively. The ERR was calculated using the following equation: ERR= 11 × effort/6 × reward. If the ERR value is >1.0, it shows that amount of effort is not rewarded adequately. In this study, the Cronbach’s alpha coefficients for the effort, reward, and overcommitment subscales were 0.879, 0.814, and 0.753, respectively.

### 2.5. Measurement of Psychological Capital (PsyCap)

PsyCap was measured using the 24-item Chinese version of the Psychological Capital Questionnaire (PCQ-24), which was developed by Luthans, and has subsequently been validated with respect to reliability and validity in multiple samples [37]. It consists of four dimensions: self-efficacy, hope, resilience, and optimism. Each of the items are scored on a 6-point Likert-type scale in which 1 indicates strong disagreement and 6 indicates strong agreement, with higher values indicating higher levels of PsyCap. The Cronbach’s alpha coefficients of self-efficacy, hope, resilience, and optimism were 0.910, 0.884, 0.765, and 0.868, respectively.

### 2.6. Statistical Analysis

Data analyses were conducted using SPSS 17.0 (SPSS China Corp., Shanghai, China) and Amos 6.0 (SPSS Inc., Chicago, IL, USA). ALL statistical tests were two-sided and the significance level was set at *p* < 0.05. Pearson’s correlation was used to examine correlations among job satisfaction, occupational stress and PsyCap. Hierarchical linear regression analysis (HLR) was calculated to explore the association among variables in relation to job satisfaction by building progressive models. Job satisfaction of Chinese township cadres was used as a dependent variable. The independent variables were entered in three steps. In step 1, the demographic characteristics of age, years of service, duties, gender, marital status, education, and monthly income were controlled as additional independent variables; in step 2, dimensions of occupational stress were added; and in step 3, PsyCap was added.

**Figure 1:**
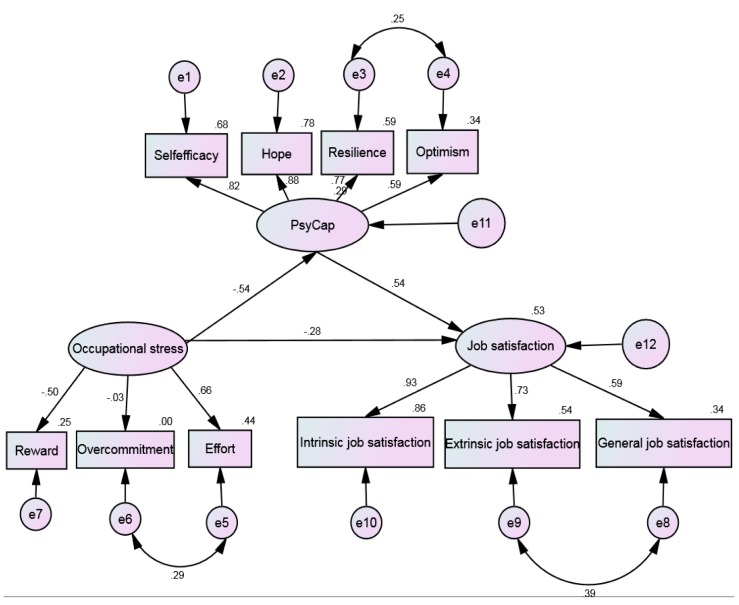
The structural equation model on the relationships between occupational stress, Job Satisfaction and Psychological Capital (PsyCap) among whole sample. e1–e10: the measurement error of each observed variable to estimate latent variable. e11–e12: the residual that may affect the endogenous latent variables except the exogenous latent variables.

Moreover, structural equation modeling (SEM), was employed to further verify hypothetical relationships based on the result of HLR among the dimensions of job stress, job satisfaction, and PsyCap. SEM with latent variables was considered a more effective statistical technique than multiple regression analysis when multiple variables are acting on an outcome and interacting with each other at the same time, thus providing greater insight into direct and indirect effects [38]. According to the HLR, and related research results, a hypothetical model was developed as shown in Figure 1. To determine whether the proposed model shows a good fit to the data, χ^2^chi-square analysis (χ^2^/df < 5), the root mean square error of approximation (RMSEA) < 0.08, goodness of fit index (GFI) > 0.90, comparative fit index (CFI), and Tucker-Lewis index (TLI) > 0.90 were utilized.

In addition, bootstrapping was performed to confirm the mediation effect. Based on a related study, bootstrapping, which involves repeatedly sampling from the data set and examines the indirect effect in each resampled data set, is an increasingly effective non-parametric method of testing the mediation effect [39]. It can calculate a bias-corrected 95% confidence interval and 95% confidence interval (95%CI) for each a × b product for each independent variable. If 95%CI excludes 0, it indicates that the mediating role is statistically significant. The bootstrap estimate was based on 2000 bootstrap samples in this study.

## 3. Results

### 3.1. Demographic Characteristics

Demographic characteristics of the subjects and the distributions of job satisfaction in categorical variables are shown in Table 1. In this study, the age of participants ranged from 19 to 64 (37.46 ± 9.32). The mean score of job satisfaction among the township cadres population was 71.21 ± 12.98.

### 3.2. Correlations between Occupational Stress, PsyCap and Job Satisfaction

The results of the Pearson correlation analyses are shown in Table 2. All dimensions of occupational stress were negatively related to intrinsic, extrinsic, and overall job satisfaction. While ERR was negatively correlated with PsyCap; PsyCap was positively related to intrinsic and extrinsic job satisfaction.

### 3.3. Associations between Occupational Stress, PsyCap and Job Satisfaction

The data were further employed for hierarchical regression analyses using job satisfaction as the explained variable, where the occupational stress and PsyCap were taken as principal predictors.

The result of hierarchical regression of job satisfaction is presented in Table 3. Each step of the independent variables made a significant contribution to variance in job satisfaction. While the demographic factors of gender, age, educational level, marital status, duties, Years of service in township, and average monthly income contributed to 1.70% of the variance in job satisfaction in step 1, the dimensions of occupational stress accounted for 14.0% of the variance in job satisfaction in step 2. It also demonstrated that ERR (β = −0.385, *p* < 0.001) and overcommitment (β = −0.109, *p* < 0.001) can predict negative job satisfaction after controlling for demographic characters. Step 3, which was established on the basis of step 2 with PsyCap added, showed that the effect of PsyCap on job satisfaction was positive and significant (β = 0.520, *p* < 0.001), accounting for an additional 24.8% in the variance of job satisfaction. It also showed that ERR (β = −0.205, *p* < 0.001) and PsyCap (β = −0.520, *p* < 0.001) were identified as significant direct predictors of job satisfaction under the control of demographic variables.

### 3.4. The Mediating Role of PsyCap in the Relationship between Occupational Stress and Job Satisfaction

Results of the SEM to verify the mediating role of PsyCap are presented in Figure 1. The data fail to support the proposed model using such evaluation indexes as χ^2^/df = 7.85, GFI = 0.966, NFI = 0.955, IFI = 0.960, CFI = 0.960, TLI = 0.942, and RMSEA = 0.072. To develop a better-fitting model, several pairs of error terms were specifically correlated according to the modification indexes. As a result, the final model shows a good fit of data χ^2^/df = 4.745, GFI = 0.983, NFI = 0.977, IFI = 0.982, CFI = 0.982, TLI = 0.972, and RMSEA = 0.050.

In the structural path model, occupational stress had a direct and significant effect on PsyCap (r = −0.54, *p* < 0.001) and job satisfaction(r = −0.28, *p* < 0.001), and PsyCaphad significant and direct effect on job satisfaction(r = 0.54, *p* < 0.001). Moreover, there still was significant and indirect effect (r = −0.293, *p* < 0.001) of occupational stress on job satisfaction with psychological capital as a mediator.

In addition, bootstrapping was performed to confirm the mediation effect. The method of bootstrapping is an increasingly popular non-parametric method for testing the mediation effect [40] that involves repeated sampling from the data set and estimates of the indirect effect in each resampled data set. Bootstrapping estimates the confidence interval (CI) for the mediation effect under most conditions. As shown in Table 4, for each independent variable, the 95%CI (−0.249, −0.341) and bias-corrected 95%CI (−0.250, −0.342) of the mediating effect (a × b product) all excluded 0, which indicated that the mediating role of PsyCap was statistically significant. We also calculated that the total effect of occupational stress on job satisfaction was 57.0% and the indirect effect accounted for 51.40% of the total effect. The bootstrap estimate presented in our study was based upon 2000 bootstrap samples (Table 4).

## 4. Discussion

Many scholars have explored the job satisfaction of professional staff, such as behavior and other occupational phenomena, in many professions, including teaching, medicine, law enforcement, and so forth [7,41]. The current study is one of the few to investigate job satisfaction among Chinese township cadres, and to examine the correlation between occupational stress and job satisfaction, and it is the first study to explore the mediating role of PsyCap in these relationships. Our research indicates that 59.74% of township cadres express an attitude of satisfaction with their jobs, and they also enjoy a moderate level of satisfaction (the mean score of job satisfaction was 71.21, SD = 12.98), which was similar with that of police officers whose job satisfaction was measured using the same scale [7].

Additionally, our research also indicates that ERR and overcommitment are negatively correlated with job satisfaction, which is consistent with previous findings among other occupations [12,42,43]. In step 2 of the regression, based on the absolute value of β, ERR and overcommitment accounted for the observed variance in job satisfaction. It is noteworthy that ERR is more strongly associated with job satisfaction than with overcommitment. The strong association between ERR and job satisfaction may be related to China’s national conditions and the nature of township cadres’ work. In China, the township cadres work in the countryside with poor economic conditions and serve for famers. Under such conditions, those who often must expend more effort to achieve the organization’s overall goals, but receive a lower reward, may experience lower levels of job satisfaction. Occupational stress has been identified as a risk factor for poor mental health and lower job satisfaction [3,13]. A previous study reported that notable sources of stress for township cadres included low reward and heavy tasks [21]. So the administrators of township cadres should balance effort and reward to increase the level of job satisfaction.

Psycap is important concept of organizational behavior. Luthans and colleagues reported that higher levels of PsyCap enhance personal confidence and increased effort to pursue success, preserve the will to achieve a goal, and increase the positive psychological capacity to deal with difficult problems [35]. Other professional groups have paid much attention to the development of PsyCap [44,45,46]. One study stated that PsyCap was considered as a positive resource to improve job satisfaction in Chinese doctors [7,47]. Moreover, a study has reported that PsyCap could moderate the association between job satisfaction and performance [48]. Our results revealed that PsyCap was significantly correlated with occupational stress and job satisfaction, which motivated us to explore the indirect effect of occupational stress in predicting job satisfaction. As far as we know, there have been no studies designed to assess the level of township cadres’ PsyCap. Our study concludes that it is important to enhance the components of PsyCap in township cadres with the purpose of improving job satisfaction.

In the current study, the major contribution indicated that job stress not only had a direct effect on job satisfaction, but also had an indirect effect on job satisfaction via PsyCap among township cadres. In other word, township cadres with higher scores of job stress may have lower PsyCap values, which in turn would lead to lower levels of job satisfaction. Practically, our findings reveal that township cadres have moderate levels of job satisfaction. It is important for township administrators in China to be aware of the levels of job satisfaction among the township cadres and for them to develop measures to increase levels of job satisfaction. According to our results, the administrative department of township cadres maybe able to decrease township cadre occupational stress, and improve PsyCap to improve the level of job satisfaction. However, it is difficult to balance effort and reward with overcommitment directly in township cadres because of the special working environment and nature of the work in townships. Our results provide a new perspective for administrators to develop the PsyCap resource to improve job satisfaction among the township cadres. Empirical studies suggest that the four components of PsyCap (self-efficacy, hope, resilience, and optimism) can be manipulated using effective measures and the psychological capital intervention (PIC) training model has been developed for this purpose [49,50,51]. Therefore, measures of enhancing PsyCap for township cadres’ in China should be developed as soon as possible.

Our results suggest that there is a great need for township administrators to develop and implement effective measures for improving job satisfaction. However, the present study does have some limitations. First, this study is a cross-sectional design, so causal conclusions cannot be drawn. The results of this study should be confirmed by a longitudinal study. Second, all participants in the present study comprised only a small proportion of all township cadres in China. Therefore, the sample may not be representative of all Chinese township cadres and the generalization of our data should be made with caution. Moreover, the current study only focused on several variables associated with job satisfaction. The direct effect of occupational stress on job satisfaction may occur via other variables that have not been explored. Future studies should be developed to measure these variables.

## 5. Conclusions

A moderate level of job satisfaction was detected among township cadres in the northeast of China. The dimensions of occupational stress (ERR and overcommitment) were negatively associated with job satisfaction, whereas PsyCap was positively associated with job satisfaction. Furthermore, PsyCap was shown to play a role in partially mediating the effect of occupational stress on job satisfaction. Interventions should be developed to improve the job satisfaction of Chinese township cadres. Importantly, since PsyCap can be measured and developed, administrators should develop interventions to enhance PsyCap.

## Figures and Tables

**Table 1:** Demographic characteristics of sample and distributions of job satisfaction (N = 1525).

Variable	N (%)	Job Satisfaction Mean (SD)
Gender		
Males	692 (45.38)	71.67 (0.52)
Females	833 (54.62)	70.82(0.43)
Age		
<30	389 (25.51)	71.15 (0.67)
30~40	579 (37.97)	70.79 (0.54)
>40	557 (36.52)	71.69 (0.55)
Marital status		
Single	291 (19.08)	70.58 (0.79)
Married/cohabitation	1163 (76.26)	71.06 (0.37)
Divorced/segregated/widow	71 (4.66)	76.15 (1.78)
Education		
College or above	763 (50.05)	71.52 (0.48)
Junior college	538 (35.29)	71.53 (0.56)
Senior high school	175 (11.48)	69.15 (0.89)
Middle school or under	49 (3.21)	70.14 (1.61)
Duties		
Senior management	20 (1.31)	73.35 (2.97)
Middle management	191 (12.52)	73.73 (0.87)
Grass-roots management	411 (26.95)	72.33 (0.67)
Ordinary employee	820 (53.77)	70.17 (0.45)
Other	83 (5.44)	69.54 (1.36)
Monthly income		
≤1500	143 (9.38)	68.53 (1.03)
1501–2500	443 (29.05)	69.86 (0.61)
2501–3500	678 (44.46)	71.95 (0.49)
≥3500	261 (17.11)	73.05 (0.81)
Years of service in township		
<3	213 (13.97)	70.80 (0.96)
3~	287 (18.82)	71.18 (0.79)
6~	325 (21.31)	71.36(0.69)
10~	700 (45.90)	71.28 (0.48)

**Table 2:** Correlation between occupational stress, PsyCap and job satisfaction.

Variable	Mean	SD	1	2	3	4	5	6
1. ERR	0.71	0.34	1					
2. Overcommitment	15.01	2.75	0.194 **	1				
3. PsyCap	101.75	18.13	−0.319 **	0.121 **	1			
4. Intrinsic job satisfaction	42.83	8.06	−0.346 **	−0.053 *	0.593 **	1		
5. Extrinsic job satisfaction	21.04	4.61	−0.332 **	−0.015	0.457 **	0.678 **	1	
6. Over job satisfaction	71.21	12.98	−0.372 **	−0.045 *	0.587 **	0.942**	0.871 **	1

Notes: ERR: effort/reward ratio; PsyCap: psychological capital; * *p* < 0.05; ** *p* < 0.001.

**Table 3:** The hierarchical linear regression analysis for job satisfaction in the total population.

Variables	Job Satisfaction		
	Step 1 (β)	Step 2 (β)	Step 3 (β)
Gender	−0.010	−0.001	0.009
Age	0.007	0.001	−0.010
Marital status 1 (Single vs. Married/Cohabitation)	0.011	0.009	−0.003
Marital status 2 (Divorced/Widow/Separated vs. Married/Cohabitation)	0.086 *	0.063 *	0.049
Monthly income 1 (≤1500 vs. ≥3500)	0.037	0.056	0.066 *
Monthly income 2 (1501–2500 vs. ≥3500)	0.111 *	0.095 *	0.082 *
Monthly income 3 (2501–3500 vs. ≥3500)	0.106 *	0.093 *	0.076
Education (Senior high school vs. Middle school or under)	−0.019	−0.045	−0.045
Education 2 (Junior college vs. Middle school or under)	0.037	−0.027	−0.082
Education 3 (college or above vs. Middle school or under)	0.035	−0.029	−0.101
Duties 1 (Senior management vs. Other)	0.022	0.005	0.059
Duties 2 (Middle management vs. Other)	−0.020	−0.015	0.041
Duties 3 (Grass-roots management vs. Other)	−0.076	−0.061	0.005
Duties 4 (Ordinary employee vs. Other )	−0.034	−0.055	−0.019
Years of service in township 1 (<3 vs. >10)	−0.023	−0.013	0.012
Years of service in township 2 (3–6 vs. >10)	−0.041	−0.005	0.019
Years of service in township 3 (6–10 vs. >10)	−0.087	−0.063	−0.035
ERR		−0.385 **	−0.205 **
Overcommitment		−0.109 **	−0.010
PsyCap			0.520 **
F	2.542 **	15.937 **	49.263 **
Adjusted R^2^	0.017	0.157	0.388
△R^2^		0.140	0.248

Notes: ERR: effort/reward ratio; PsyCap: psychological capital; * *p* < 0.05; ** *p* < 0.001 (two-tailed).

**Table 4:** Mediating role of PsyCap on the associations between occupational stress and job satisfaction.

Variables	Estimates	Bootstrap	
		Bias-Corrected 95%CI	
		Lower Bounds	Upper Bounds
Occupational stress→Job satisfaction (Total effects)	−0.570	−0.500	−0.645
Occupational stress→Job satisfaction (Indirect effects)	−0.293	−0.250	−0.342
Occupational stress→Job satisfaction (Direct effects)	−0.277	−0.192	−0.366
